# Transplantation of Human iPS Cell-derived Cerebral Cortical Neurons Promotes Fine Motor Recovery in a Female Mouse Model of Ischemic Stroke

**DOI:** 10.1007/s12015-025-10981-x

**Published:** 2025-10-11

**Authors:** Hokuto Yamashita, Tetsuhiro Kikuchi, Yusaku Kodaka, Daisuke Doi, Megumi Ikeda, Jun Takahashi

**Affiliations:** 1 Department of Clinical Application, Center for iPS Cell Research and Application, Kyoto University, Kyoto, Japan; 2 Department of Neurosurgery, Kyoto University Graduate School of Medicine, Kyoto, Japan; 3 Kobe Research Center, RACTHERA Co., Ltd., Hyogo, Japan

**Keywords:** IPS cell, Cerebral organoid, Ischemic stroke, Cell transplantation

## Abstract

**Background:**

Stroke is a leading global health concern, with cerebral infarction accounting for 62% of cases. Despite advances in acute-phase treatments, functional impairments such as motor deficits remain prevalent. This study investigates the potential of human induced pluripotent stem cell (iPSC)-derived cerebral cortical neurons for neural regeneration and motor function recovery in a female mouse model of ischemic stroke.

**Methods:**

Cerebral infarction was induced using the Rose Bengal photothrombosis method, followed by transplantation of iPSC-derived cortical neurons into the area adjacent to the infarction. Behavioral recovery was assessed using the foot fault and cylinder tests. Histological analysis was performed to evaluate graft integration and neurite extension.

**Results:**

Foot fault test demonstrated significant improvements in fine motor function in the transplantation group compared to the vehicle group. However, no recovery was observed in the cylinder test, which assesses gross motor function. Neurite extension from grafted cells was observed along the corticospinal tract, with axonal projections reaching the spinal cord in 68% of transplanted mice. In addition, neurite outgrowth extended to the thalamus, superior colliculus, and vestibular nucleus, suggesting integration into multiple neural circuits. Histological analysis revealed that 16.4% and 47.3% of grafted cells expressed CTIP2 and SATB2, respectively, indicating the presence of both deep- and upper-layer cortical neurons.

**Conclusions:**

This study demonstrates that iPSC-derived cortical neurons extend axons along the corticospinal tract and can promote fine motor recovery after stroke. However, further research is needed to validate functional connectivity and long-term safety. These findings offer a promising avenue for developing cell-based therapies for stroke patients.

**Graphical Abstract:**

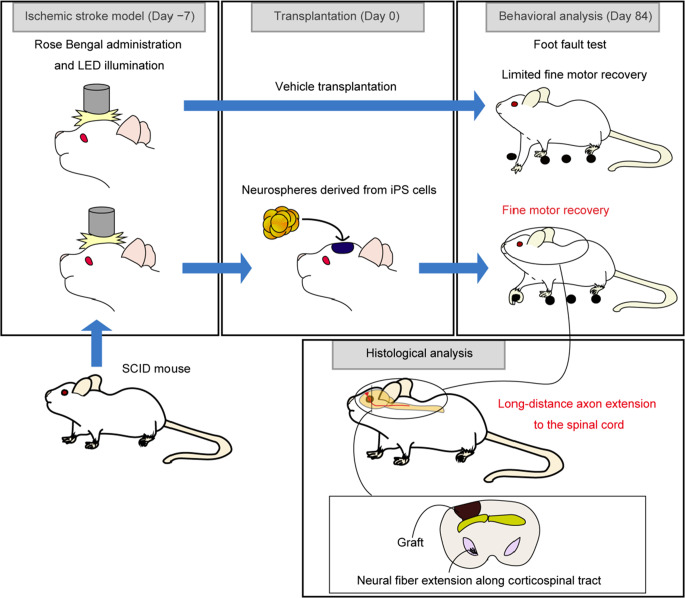

## Background

Stroke represents a significant global health challenge, as highlighted by the World Stroke Organization’s Global Stroke Fact Sheet 2022, which reports an annual incidence of 12.2 million new cases and 6.5 million deaths. Cerebral infarction, accounting for 62% of all strokes, affects approximately 7.6 million individuals annually [1]. Common sequelae include fine motor impairment, dysarthria, and paralysis, all of which severely impact daily living. Although advances in acute-phase treatment have reduced cerebral infarction-associated mortality, the prevalence of sequelae and functional impairments remains substantial. Consequently, there is an urgent need for therapies promoting functional recovery and neural regeneration post-cerebral infarction.

Cell transplantation therapy has emerged as a promising approach for treating cerebral infarction, with the potential to facilitate neural circuit reconstruction and functional recovery. Various cell types, including mesenchymal and neural stem cells, have been investigated for their potential in stroke treatment. However, challenges such as poor long-term graft survival, limited functional integration, and inconsistent behavioral recovery outcomes persist [2–6]. While some studies have demonstrated that transplanted neurons can extend axons and establish synaptic connections, the extent to which these connections translate into meaningful motor recovery remains unclear.

Recent advancements in pluripotent stem cell technology have facilitated the creation of cerebral organoids— three-dimensional brain model tissues closely mimicking the human brain’s complex neural circuitry and cellular organization [7]. These organoids serve as a promising source of cortical neurons for transplantation and have been shown to survive and extend neurites following grafting [8, 9]. Several studies have reported successful cell engraftment and neurite extension, but whether these transplants can effectively restore motor function following brain damage remains unclear [10–14]. Here, we transplanted human induced pluripotent stem cell (iPSC)-derived cerebral organoids into a female mouse cerebral infarction model to evaluate neurological behavior and neurite extension from grafts.

## Methods

### Maintenance of iPSCs

A human iPSC line (S17) was established from peripheral blood mononuclear cells purchased from Lonza (Basel, Switzerland) using Sendai virus vectors [15]. The iPSCs were cultured on iMatrix (Matrixome, Osaka, Japan) in StemFit medium (Ajinomoto, Tokyo, Japan). For passaging, iPSCs were dissociated into single cells using 0.5× TrypLE Select (Thermo Fisher Scientific, Waltham, MA, USA) with 250 µM EDTA for 8 min and re-plated onto 6-well plates at a density of 1–1.5 × 10^4^ cells per well in StemFit medium supplemented with 10 µM Y-27,632 (FUJIFILM Wako Pure Chemicals, Oaksa, Japan). Cells were passaged every 7 days, with the medium changed on days 2, 4, 5, and 6 following passaging.

### Differentiation of Human iPSCs

**Fig. 1:**
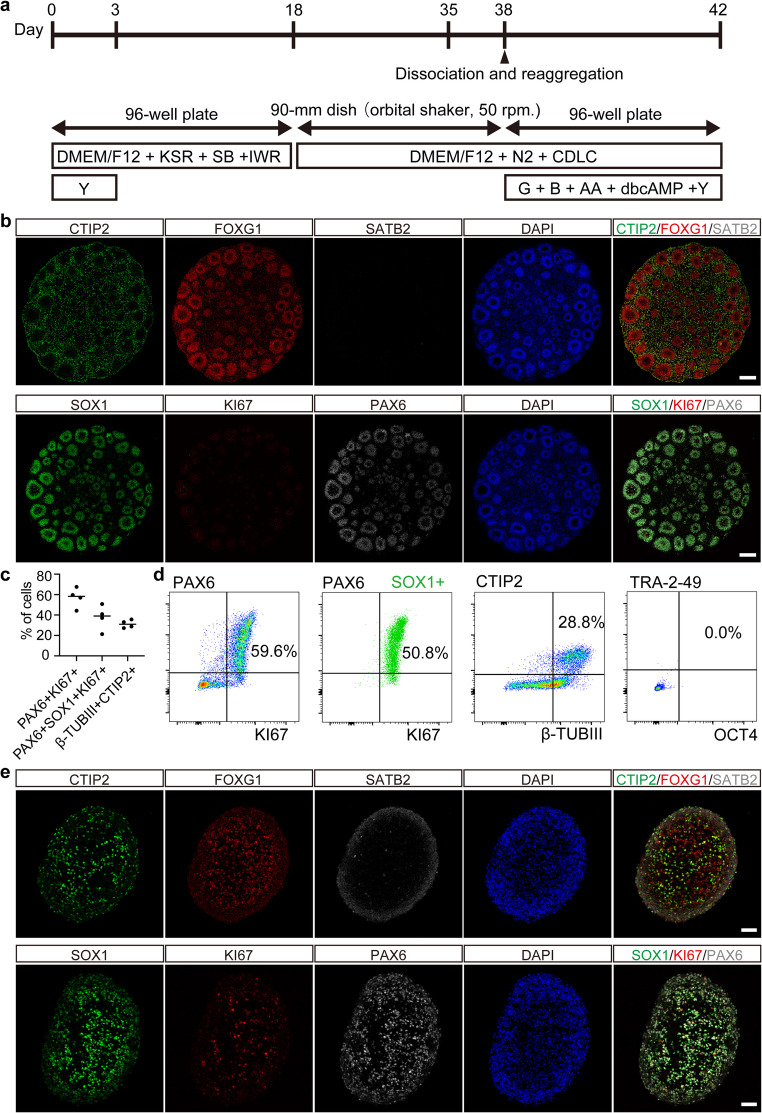
Generation and characterization of human iPSC-derived cortical organoids and neurospheres. (**a**) Schematic representation of the differentiation protocol used to generate cerebral cortical neurons from human iPSCs. iPSCs were differentiated into cerebral organoids, which were then dissociated and reaggregated into neurospheres for transplantation. KSR: knockout serum replacement, SB: SB431542 (a transforming growth factor β inhibitor), IWR: IWR1e (a Wnt inhibitor), Y: Y-27,632 (a ROCK-inhibitor), CDLC: chemically defined lipid concentrate, G: glial cell-line derived neurotrophic factor, B: brain-derived neurotrophic factor, dbcAMP: Dibutyryl cyclic adenosine monophosphate (**b**) Representative immunostaining images of organoids at day 35 of differentiation. Rosette-like structures positive for neural progenitor markers FOXG1, SOX1, and PAX6 are evident. Surrounding cells express the deep-layer cortical neuron marker CTIP2, while the upper-layer marker SATB2 is absent. Scale bar: 200 μm. (**c**) Quantification of the cellular composition in dissociated neurospheres at day 41 by flow cytometry. Data are presented as mean ± S.D. (*n* = 4 independent differentiation batches). (**d**) Representative flow cytometry plot for the markers quantified in (c), showing populations of proliferating cortical progenitors (PAX6^+^/SOX1^+^/KI67^+^) and deep-layer neurons (CTIP2^+^/β-TUBIII^+^). (**e**) Representative immunostaining images of neurospheres at day 42, showing uniform expression of neuronal markers. Scale bar: 50 μm

Human iPSC-derived organoids were generated using a modified SFEBq technique (Fig. 1a) described by Kitahara et al. [9]. One day before initiating differentiation, iPSCs were treated with StemFit medium without solution C supplemented with 5 µM SB431542 (a transforming growth factor β inhibitor, TOCRIS Bioscience, Bristol, UK). For dissociation, iPSCs were treated with 0.5× TrypLE Select containing 250 µM EDTA for 10 min to optimize feeder-free culture conditions.

Dissociated cells were then plated onto low-cell adhesion V-bottom 96-well plates (PrimeSurface MS-9096 V, Sumitomo Bakelite, Tokyo, Japan) in the differentiation medium containing 50 µM Y-27,632 at a density of 9,000 cells per well. The differentiation medium comprised DMEM/F-12 GlutaMAX (Thermo Fisher Scientific, Waltham, MA, USA) supplemented with 20% (v/v) KnockOut Serum Replacement (KSR), 5 µM SB431542, and 3 µM IWR1e (a Wnt inhibitor, Calbiochem). Half of the medium was replaced every three days until day 15. SFEBq culture was initiated on day 0.

On day 18, cell aggregates were transferred to 90-mm non-coated dishes (MS-1390R, Sumitomo Bakelite, Tokyo, Japan) and cultured in DMEM/F-12 GlutaMAX supplemented with 1% (v/v) N-2 Supplement (Thermo Fisher Scientific, Waltham, MA, USA), 1% (v/v) Chemically Defined Lipid Concentrate (CDLC; Thermo Fisher Scientific, Waltham, MA, USA), 0.25 µg/ml Amphotericin B (Thermo Fisher Scientific, Waltham, MA, USA), 100 U/ml penicillin, and 100 µg/ml streptomycin. Organoids were cultured on an orbital shaker at 50 rpm in an atmosphere of 20% O_2_ and 5% CO_2_ to prevent the need for 40% O_2_. Complete medium changes were performed once every 3 or 4 days from day 18 to day 38.

On day 38, organoids were dissociated with 0.5× TrypLE Select with 250 µM EDTA for 10 min, followed by replating onto low-cell adhesion V-bottom 96-well plates at a density of 30,000 cells per well to make neurospheres. Freshly prepared neurospheres were transplanted without being frozen.

### Flow Cytometry

Flow cytometric analysis was performed on day 41. Dissociated cells were stained by the LIVE/DEAD^®^ Fixable Dead Cell Stain Kit (Thermo Fisher Scientific, Waltham, MA, USA) and FITC-conjugated anti-TRA-2–49 antibody (Merck, FCMAB133F), followed by fixation by 4% paraformaldehyde (PFA) for 30 min. After fixation, cells were permeabilized by Perm/Wash buffer (BD Biosciences) for 30 min, stained by antibodies for intracellular staining, and rinsed with Perm/Wash buffer. Primary antibody used was as follows: PAX6 (Alexa647-conjugated PAX6, BD Biosciences, 562249), KI67 (Alexa488-conjugated KI67, BD Biosciences, 561165), SOX1 (PerCP-Cy5.5-conjugated SOX1, BD Biosciences, 561549), OCT4 (Alexa647-conjugated OCT4, BD Biosciences, 560329), CTIP2 (FITC-conjugated, Abcam, ab123449), β-TUBIII (Alexa647-conjugated TUJ1, BD Biosciences, 560394). Stained samples were analyzed using a FACSAria III flow cytometer (BD Biosciences, San Jose, CA, USA).

### Animals

All animal procedures were conducted in accordance with the Guidelines for Animal Experiments of Kyoto University and the Guide for the Care and Use of Laboratory Animals (Institute of Laboratory Animal Resources, Washington, DC). Twelve-week-old female SCID mice (C.B-17/Icr-scid/scidJcl, Clea Japan, Inc., Tokyo, Japan) were used in this study. Female mice were selected for primary studies due to a higher survival rate than male mice after cerebral infarction induction. Mice were housed under a 12-hour light/dark cycle with free access to food and water.

### Creation of Ischemic Stroke Model in Mice

Ischemic stroke models were created using the Rose Bengal photothrombosis method. Mice were anesthetized with an intraperitoneal injection of a mixture of medetomidine hydrochloride (0.75 mg/kg), midazolam (4 mg/kg), and butorphanol (5 mg/kg), and a skin incision was made to expose the skull at the site for cerebral infarction induction. Rose bengal was administered intraperitoneally at a dose of 10 µL/g. Ten minutes post-administration, the skull was illuminated for 10 min using an LED illumination device (CL6000 LED, Zeiss, Oberkochen, Germany). The illuminated region encompassed the rostral and caudal forelimb motor cortex, spanning from 2.0 mm caudal to 4.0 mm rostral to the bregma and from 0.5 mm to 3.5 mm lateral from the midline. Animals in which no cerebral infarction was observed on MRI were excluded.

### Transplantation of iPSC-derived Neurospheres

One week after stroke induction, mice were anesthetized again using the same three-anesthetic mixture. Neurospheres containing 1.5 × 10^5^ cells/µL were prepared for transplantation. A craniotomy approximately 2 mm in diameter was performed at coordinates 2 mm lateral and 1 mm rostral to the bregma. A transplantation syringe was inserted 2 mm deep into the brain, then withdrawn 1 mm before delivering 1 µL of neurospheres at a rate of 0.1 µL/s. The syringe was left in place for 1 min before being withdrawn. Forty-seven animals were assigned to the transplantation group and received cell transplantation, while 24 were assigned to the vehicle group and were injected with the same medium used for cell transplantation. Randomization was not performed; however, to minimize allocation bias, surgeries for the cell administration group and the vehicle group were performed alternately by the same operators.

### Behavioral Analysis

Two behavioral tests were conducted before cerebral infarction and every two weeks post-infarction to assess motor function recovery. All behavioral analyses were performed by observers who were blinded to the experimental groups.

#### Foot Fault Test

Mice were placed on an 11 mm × 11 mm wire mesh grid. The number of times the mice’s forelimbs slipped through the mesh during a 3-minute walk was recorded as faults. The fault ratio was calculated as the number of faulty steps divided by the total number of steps. Asymmetry between the left and right limbs was assessed using the formula: (number of faulty steps)/(total number of steps), with the difference between the left and right sides used to determine the asymmetric score [16].

#### Cylinder Test

Mice were placed in a transparent cylinder with a diameter of 15 cm and a height of 28 cm. The paw that first touched the cylinder wall during rearing from an initial position with both paws on the floor was recorded. The test lasted for 10 min or until 20 trials were completed. The asymmetric index was calculated as (number of right paw touches)/(number of right paw touches + number of left paw touches) [17].

### Magnetic Resonance Imaging (MRI)

Intracranial MRI was performed on mice within 7 days following the induction of cerebral infarction. Mice were anesthetized using the same mixture of three anesthetics as stroke model creation. T2-weighted MRI images (TR = 2000 ms, TE = 55 ms, slice thickness = 1 mm) were obtained using a 0.5-tesla MRI scanner (Bruker BioSpin GmbH, Rheinstetten, Germany).

### Euthanasia and Perfusion Fixation

Animals that became debilitated during the observation period were euthanized and dissected. No animals were found to have died due to the effects of the graft. Healthy animals were observed for 12 weeks. Of the 77 animals, 71 were included in the final analysis. After the observation period, the mice were euthanized with pentobarbital (300 mg/kg) and perfused transcardially with 4% paraformaldehyde. Brains were post-fixed in 4% paraformaldehyde for 24 h and then transferred to a 30% sucrose solution for cryoprotection. Brain sections were sliced at a thickness of 40 μm using a cryostat and stored in an antifreeze solution at − 30 °C until further processing.

### Immunostaining

For in vitro analyses, brain organoids or neurospheres were frozen and sectioned at a thickness of 10 μm using a cryostat. Fluorescence immunostaining was performed as follows. Brain sections were incubated in Target Retrieval Solution (pH 6) (Agilent) at 75 °C for 15 min to facilitate antigen retrieval. The following primary antibodies and dilutions were used: CTIP2 (Abcam, ab18465, 1:500), FOXG1 (TAKARA, M227, 1:500), KU80 (Abcam, ab80592, 1:500), and Human Nucleus (Merck, MAB1281, 1:500), SATB2 (Abcam, ab51502, 1:200), and SOX1 (R&D, AF3369, 1:200). The next day, sections were incubated with secondary antibodies, diluted at 1:400, and then mounted. For 3,3’-Diaminobenzidine Tetrahydrochloride (DAB) staining, sections were treated with 0.3% hydrogen peroxide (H_2_O_2_) for 15 min and incubated overnight with the primary antibody against human NCAM (Santa Cruz, sc-106) at a 1:1000 dilution. A biotin-conjugated secondary antibody, diluted at 1:1000, was used, followed by the application of A and B solutions. The DAB reaction was performed with a concentration of 10 mg/ml. After the reaction, sections were counterstained with hematoxylin and mounted.

### Image Analysis

For graft volume measurement, the DAB-stained area in one in six sections was measured using ImageJ software. The volume was calculated by multiplying the measured area by the section thickness. For cell counting, images were captured using a confocal microscope (CQ1, YOKOGAWA, Tokyo, Japan) and analyzed with Cell Path Finder software.

### Statistical Analysis

All statistical analyses were conducted using GraphPad Prism (GraphPad Software Inc., La Jolla, CA, USA). Data are presented as mean ± standard deviation (S.D.). An a priori power analysis was not performed. The larger sample size in the transplantation group was chosen to ensure sufficient statistical power for the planned correlational analyses between histological and behavioral data. The sample size for the vehicle group was deemed sufficient for detecting differences in the primary behavioral endpoints based on previous studies. The work has been reported in line with the ARRIVE guidelines 2.0.

## Results

### Induction of Cerebral Cortical Neurons Via Organoids

Cerebral organoids were generated from iPSCs using a method based on SFEBq (Fig. 1a) [9]. Immunostaining conducted on day 35 revealed rosette-like structures, indicating the presence of neural progenitor cells, as evidenced by the expression of FOXG1, SOX1, and PAX6. Cells surrounding these structures expressed CTIP2, a marker for cortical deep-layer neurons, while SATB2-positive cells, indicative of cortical upper-layer neurons, were absent (Fig. 1b). Organoids containing only rosette-like structures were selected, dissociated on day 38, and reaggregated to form neurospheres. This procedure facilitated the production of a homogeneous population of cortical neurons. Flow cytometry analysis on day 41 showed that 60.6 ± 10.2% of the cells expressed the proliferation marker KI67, and 37.5 ± 12.2% were triple-positive for PAX6, SOX1, and KI67, identifying them as cortical neuron progenitor cells. Additionally, 31.2 ± 3.9% of cells were double-positive for the neuronal markers β-TubIII and CTIP2, corresponding to cortical deep-layer neurons. No OCT4- nor Tra-2-49-positive cells were detected (Fig. 1c, d). Immunostaining on day 42 confirmed the uniform structure of neurospheres (Fig. 1e).

### Establishment of a Stroke Model and Cell Transplantation

**Fig. 2:**
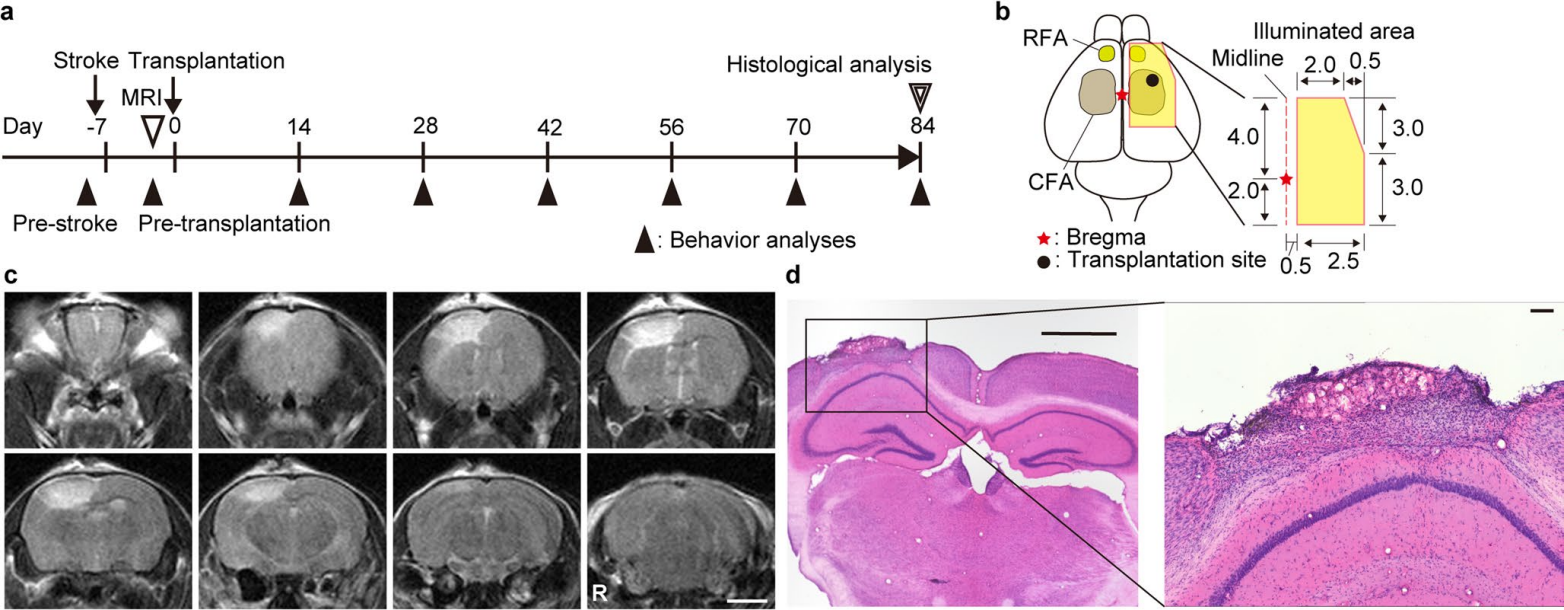
Photothrombotic stroke model creation and histological confirmation. (**a**) Schematic diagram illustrating the experimental timeline. Ischemic stroke was induced at Day − 7, followed by cell transplantation at Day 0. Behavioral analyses were conducted every two weeks for a total of 12 weeks. (**b**) Dorsal view of the mouse skull, indicating the region illuminated for stroke induction, which encompasses the rostral and caudal forelimb motor areas. The coordinates for cell transplantation are marked with a black dot. RFA: rostral forelimb area, CFA: caudal forelimb area. (**c**) Representative T2-weighted MRI images of a mouse brain taken 7 days after stroke induction. The infarcted area is visible as a hyperintense signal in the right cerebral cortex. Scale bar: 2 mm. (**d**) Representative images of Hematoxylin and Eosin (H&E) stained coronal brain section from an infarcted brain. The images show significant cortical tissue loss and atrophy in the infarct region. Scale bars: 1 mm (left) and 100 μm (right)

Cerebral infarction was induced in 12-week-old female mice using the Rose Bengal photothrombosis method on Day − 7 (Fig. 2a). The illuminated area ranged from 2 mm posterior to 4 mm anterior to the bregma in the right hemisphere, encompassing the caudal and rostral forelimb areas (Fig. 2b). One week post-infarction, the infarct region appeared as a high-intensity signal on MRI T2-weighted images, corresponding to the illuminated area. The infarct extended approximately 1.5 mm from the brain surface, indicating that all layers of the cerebral cortex were affected (Fig. 2c). The average infarct volume, calculated from MRI images, was 22.1 ± 11.4 mm³ (*n* = 71). Hematoxylin and eosin (H&E)-stained section 4 weeks post-infarction revealed cortical atrophy and scar tissue formation at the infarction site (Fig. 2d). Day 42 neurospheres, comprising 1.5 × 10^5^ cells, were transplanted seven days post-infarction (Day 0) using a stereotactic apparatus. The coordinates were 2 mm lateral to the bregma, 1 mm anteriorly, and 1 mm ventral from the brain surface (Fig. 2b).

### Cell Transplantation Improved Fine Motor Function after Stroke

**Fig. 3:**
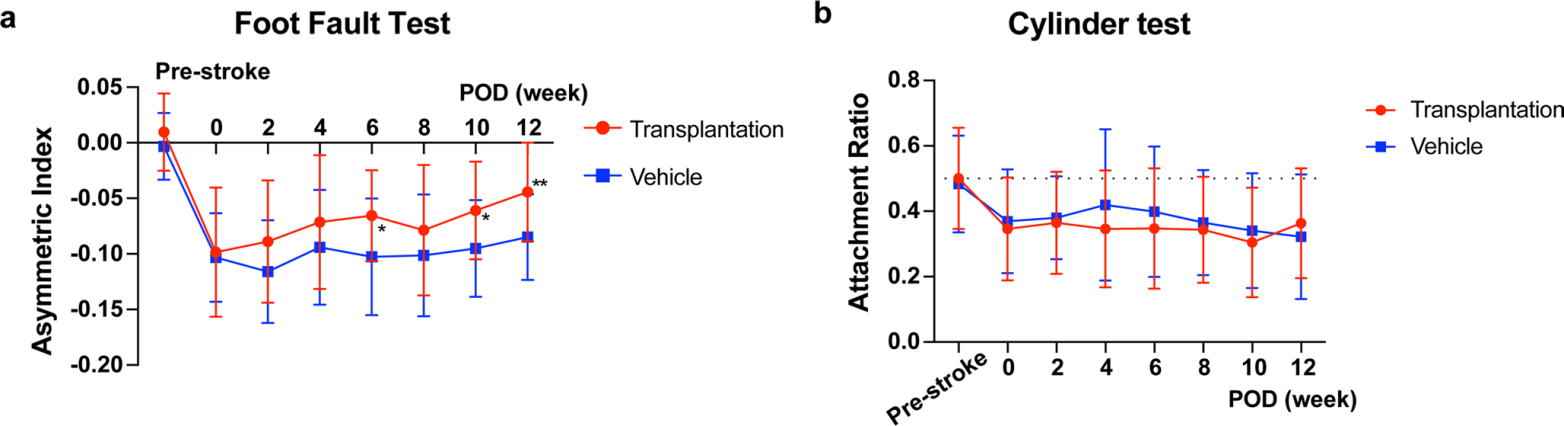
Transplantation of iPSC-derived cortical neurons improves fine motor function. (**a**) Time course of the asymmetric index in the foot fault test. The transplantation group showed a significant improvement in fine motor control compared to the vehicle group at 6, 10, and 12 weeks post-transplantation. (**b**) Attachment ratio in the cylinder test, showing the ratio of contralateral (left) paw use during vertical exploration. No significant recovery or difference between the transplantation and vehicle groups was observed throughout the study period. Data in both panels are presented as mean ± S.D. (Transplantation group, *n* = 47; Vehicle group, *n* = 24). Statistical analysis was performed using a two-way ANOVA with Tukey’s multiple comparison test (*: *p* < 0.05, **: *p* < 0.01)

The foot fault test was used to assess fine motor function, while the cylinder test evaluated gross motor function over a twelve-week period. In the foot fault test, following cerebral infarction, the fault rate increased on the side contralateral to the infarct, and a disparity in success rates between the left and right sides became apparent. The cell transplantation group (*n* = 47) exhibited a gradual improvement in motor function compared to the vehicle group (*n* = 24) and a significant improvement was observed at twelve weeks (Fig. 3a). In the cylinder test, both groups of mice also exhibited impairments following cerebral infarction (Fig. 3b). However, no significant behavioral improvements or differences between the transplanted and vehicle groups were observed over the twelve-week follow-up period.

### Grafted Cells Survived and Matured in the Host Brain

**Fig. 4:**
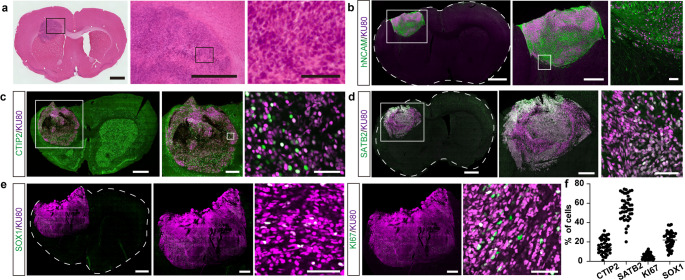
Histological analysis reveals survival, maturation, and composition of grafts at 12 weeks post-transplantation. (**a**) Representative Hematoxylin and Eosin (H&E) staining of a coronal brain section showing a well-integrated graft at the transplantation site. Scale bars: 1 mm (left), 500 μm (middle) and 50 μm (right). (**b**-**e**) Immunostaining images of a representative mouse brain graft for hNCAM/KU80 (**b**), CTIP2/KU80 (c), SATB2/KU80 (d), SOX1/KU80, SOX1/KU80 (e, left) and Ki67/KU80 (e, right). Dotted lines designate the contour of brain sections. Scale bars: 1 mm (left), 500 μm (middle) and 50 μm (right). (**f**) Quantification of the cellular composition within the grafts. The graph shows the percentage of CTIP2^+^, SATB2^+^, KI67^+^, and SOX1^+^ cells within human nuclei-positive cells in the graft. Data are presented as mean ± S.D. (*n* = 47 mice)

H&E staining of grafts twelve weeks post-transplantation confirmed successful engraftment of transplanted cells at the transplantation site (Fig. 4a). No evidence of tumor formation or teratomas was observed in any of the transplanted animals at the 12-week endpoint. The graft volume was measured at 13.5 ± 8.0 mm³ (*n* = 47). Immunohistochemical analysis revealed that the number of human-derived cells stained with the human nuclear marker KU80 was 7.6 ± 4.7 × 10^7^. Of the human-derived cells, 16.4 ± 15.2% expressed CTIP2, and 47.3 ± 27.2% expressed SATB2. Additionally, 22.2 ± 15.8% of cells were positive for the early neural differentiation marker SOX1, while 6.9 ± 5.8% expressed the proliferation marker KI67 (Fig. 4b–f).

### Grafted Neurons Extended long-distance Axons along the Corticospinal Tract

**Fig. 5:**
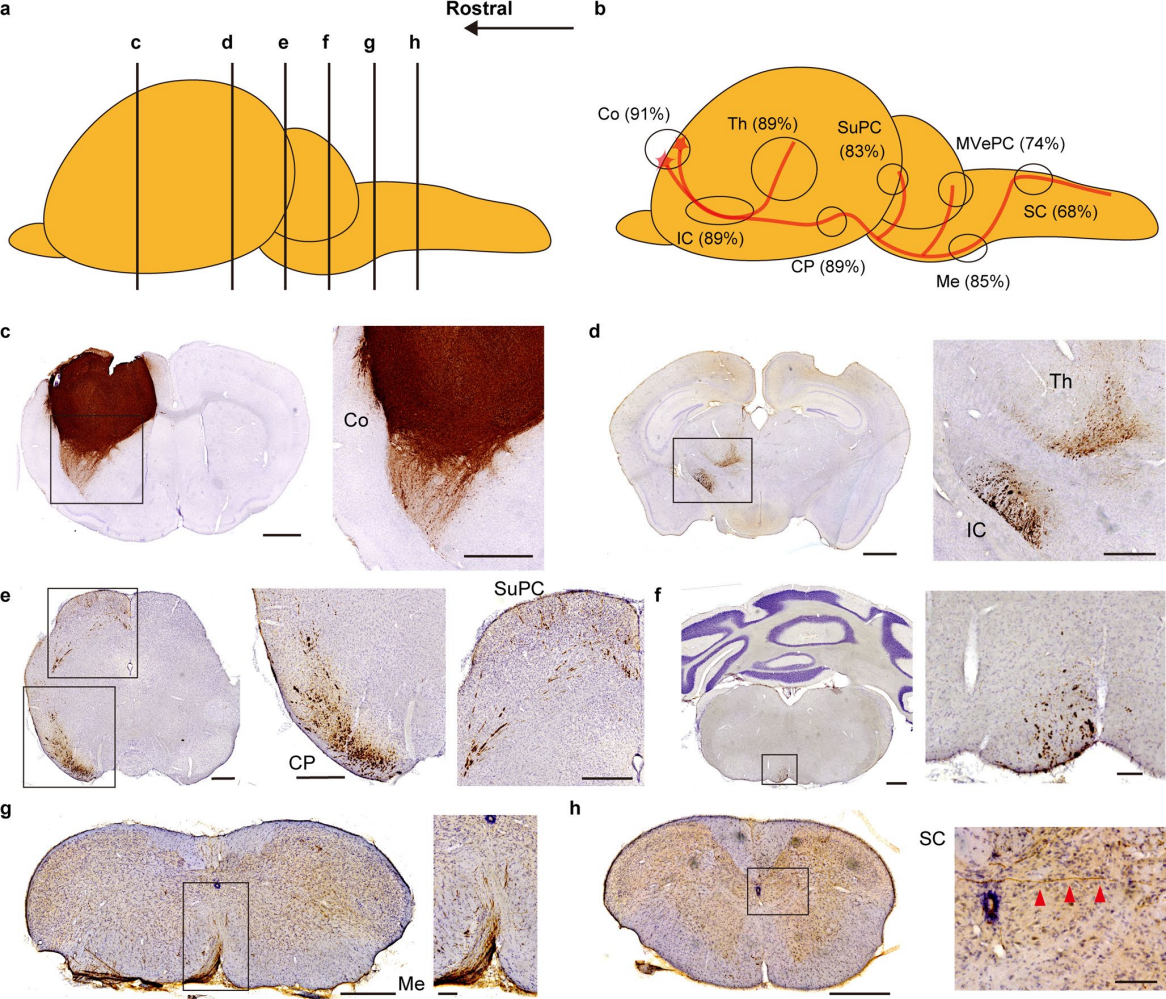
Grafted neurons extend long-distance axonal projections along major anatomical pathways. (**a**) Schematic diagram of sagittal brain sections indicating the approximate rostrocaudal levels of the images presented in (c-h). (**b**) Quantification of the percentage of transplanted mice (*n* = 47) in which graft-derived neurites, visualized by hNCAM staining, reached various target regions in the brain and spinal cord. Co: cerebral cortex, IC: internal capsule, Th: thalamus, CP: cerebral peduncle, SuPC: superior colliculus, MVePC: parvocellular part of the medial vestibular nucleus, Me: medulla, SC: spinal cord. (c-h) Representative images of hNCAM-stained sections at different anatomical levels, demonstrating robust axonal extension from the graft. Projections are visible in the ipsilateral cortex (**c**), internal capsule and thalamus (**d**), cerebral peduncle and superior colliculus (**e**), pons (**f**), medulla at the level of pyramidal decussation (**g**) and spinal cord (**h**). Scale bars: 1 mm (c, d [left]); 500 μm (d [right], e, f [left], g [left], h [left]); and 100 μm (f [right], g [right], h [right])

**Fig. 6:**
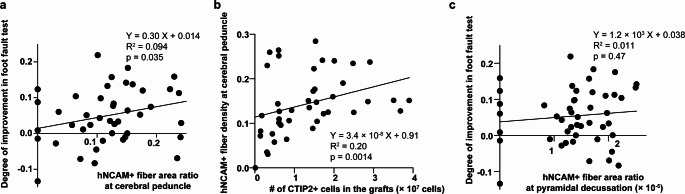
Correlation Between Histological Findings and Behavioral Improvements. (**a**) A significant positive correlation between the hNCAM^+^ fiber area ratio (hNCAM^+^ area divided by the whole area) in the cerebral peduncle and improvement in the foot fault test, thus indicating that greater neurite extension is associated with better motor function recovery. (**b**) A significant positive correlation between the number of CTIP2^+^ cells in the grafts and the hNCAM^+^ fiber area ratio in the cerebral peduncle, suggesting that a higher proportion of deep-layer neurons contributes to more robust neurite outgrowth. (**c**) Lack of a significant correlation between the hNCAM^+^ fiber area ratio in the medulla (at the level of the pyramidal decussation) and the improvement in the foot fault test. Each dot represents an individual animal (*n* = 47). Lines indicate the best-fit linear regression, and the R² and p-values were determined using the Pearson correlation coefficient

Neurite extension derived from the graft was evaluated using human-specific neural cell adhesion molecule (hNCAM) staining. Among animals receiving cell transplants, 89% exhibited axonal extension along the CST, and in 68% of cases, this extension reached the spinal cord (Fig. 5a–h). In addition to the CST, neurite extension to the ipsilateral thalamus, superior colliculus, and parvocellular part of the medial vestibular nucleus (MVePC) was observed in 89%, 83%, and 74% of transplanted animals, respectively (Fig. 5b). Notably, only a few graft-derived fibers were observed in the red nucleus. Following confirmation of neurite extension, we assessed the correlation between neurite outgrowth and behavior outcomes. A significant positive correlation was identified between hNCAM^+^ fiber area ratio (hNCAM^+^ area divided by the whole area) in the cerebral peduncle and the rate of behavioral improvement in the foot fault test (Fig. 6a). Notably, a positive correlation was also observed between the number of CTIP2-positive cells in the graft and hNCAM^+^ fiber area ratio in the cerebral peduncle (Fig. 6b). On the other hand, no positive correlation was confirmed between hNCAM^+^ fiber area ratio in the medulla at the level of pyramidal decussation and behavioral improvement (Fig. 6c).

## Discussion

Cell transplantation therapy for cerebral infarction has been extensively studied, with several clinical trials evaluating its potential efficacy [18]. However, relatively few studies have specifically investigated the capability of transplanted cells to reconstruct neural circuits [14, 19]. In this study, we transplanted human iPSC-derived cerebral cortical neurons into a female mouse model of ischemic stroke and observed neurite extension along the CST. Furthermore, animals that received transplants demonstrated significant improvements in fine motor function, as assessed by the foot fault test. Histological analysis revealed that functional recovery correlated with neurite outgrowth from the graft and the presence of CTIP2-positive neurons, suggesting that the transplanted neurons contribute to CST reorganization.

One of the key findings of this study was the post-transplantation maturation of grafted neurons. Initially, 60.6 ± 10.2% of donor cells were Ki67-positive, indicating active proliferation, while post-transplantation analysis revealed a significant reduction in proliferating cells (6.9 ± 5.8%). The proportion of CTIP2-positive cells decreased from 31.2 ± 3.9% before transplantation to 16.4 ± 15.2% afterward. Notably, while the donor population contained no SATB2-positive cells, 47.3 ± 27.2% of grafted cells were SATB2-positive after transplantation. These findings are consistent with normal brain development, in which CTIP2-positive deep-layer neurons are generated first, followed by SATB2-positive upper-layer neurons. While CTIP2-positive neurons typically project to subcortical regions such as the basal ganglia and spinal cord, SATB2-positive neurons form cortical-cortical connections via callosal projections [20, 21]. The survival of CTIP2-positive neurons is therefore critical for CST reconstruction. Our findings suggest that although postmitotic CTIP2-positive neurons are essential for CST reorganization, their survival following transplantation remains a challenge. We acknowledge that the identification of these cells as bona fide corticospinal motor neurons based on CTIP2 expression alone is a limitation. Future studies should incorporate a broader panel of molecular markers and physiological assessments to confirm their precise subtype identity. Meanwhile, the presence of proliferative progenitors poses a risk of tumorigenesis, necessitating optimized differentiation strategies to balance safety and efficacy in transplantation. Although no tumors were observed in the 12-week duration of our study, long-term tumorigenicity studies are critical. Future therapeutic applications may require more stringent purification of postmitotic neurons, for instance by fluorescence-activated cell sorting, to mitigate this risk.

Another key finding of this study is the significant improvement in fine motor function following transplantation, as evidenced by the foot fault test. Despite these gains, no significant recovery was observed in gross motor function, as assessed by the cylinder test. This discrepancy highlights a key challenge in translating preclinical findings to clinical applications. The foot fault test assesses skilled limb placement, which may not fully represent the broad-spectrum motor deficits, such as impaired gait or postural control, seen in human stroke patients. Therefore, the clinical significance of our findings should be interpreted with caution. From a neuroanatomical perspective, this test-specific recovery may be explained by previous reports indicating that spontaneous recovery in rodent stroke models primarily relies on compensatory pathways rather than true corticospinal repair [22–25] and that cortical pathways play a limited role in spontaneous post-stroke recovery [22]. The limited behavioral recovery observed in our study may be due to insufficient engagement of extrapyramidal motor pathways, such as the rubrospinal and reticulospinal tracts, which are essential for gross motor function recovery [24, 26, 27]. While significant neurite extension was observed along the CST, only a few fibers projected to the red nucleus, suggesting minimal involvement of rubrospinal circuits. Future studies should not only investigate strategies to enhance connectivity with these alternative motor pathways but also incorporate a wider range of behavioral tests that model different aspects of human motor function to maximize functional recovery.

Interestingly, we also observed neurite extension to the contralateral cerebral cortex, superior colliculus, and thalamus, consistent with normal anatomical projections [28]. These findings suggest that transplanted neurons respond to host brain signals to reconstruct multiple neural circuits. However, whether these newly formed connections contribute to functional recovery remains unclear. Crucially, our study provides only correlational data and lacks direct evidence of functional synaptic integration between grafted neurons and host circuitry. Future investigations using trans-synaptic tracing, optogenetics, and in vivo electrophysiology will be necessary to elucidate their functional impact and confirm synaptic efficacy [12]. Furthermore, while the observed projections largely follow expected anatomical pathways, the potential for aberrant or maladaptive connectivity cannot be excluded. Inappropriate connections could interfere with existing circuits or lead to unintended side effects. Long-term assessment of both functional outcomes and potential adverse effects is therefore essential for the clinical translation of this approach.

While our study highlights the potential of iPSC-derived neurons for stroke recovery, several limitations must be considered. The 12-week observation period may have been insufficient to fully capture neuronal integration and functional maturation. Additionally, sex-based differences in stroke pathology and recovery could have influenced the results, as previous studies predominantly used male rodents [12, 14]. Our study used only female mice due to higher post-surgical survival rates in our model. However, given the well-documented influence of sex on stroke outcomes and neural plasticity, this focus on a single sex limits the generalizability of our findings. Future studies must include both sexes to ensure the broader applicability and translatability of this therapeutic strategy. Furthermore, the variability in neurite extension patterns among individuals suggests that transplantation outcomes may be influenced by graft-host interactions that are not yet fully understood.

## Conclusion

In summary, our findings demonstrate that human iPSC-derived cortical neurons can integrate into stroke-affected brain regions, extend axons along the CST, and contribute to motor recovery. However, further research is required to optimize transplantation protocols, enhance functional integration, and assess long-term safety. Addressing these challenges will be crucial for establishing neuronal transplantation as a viable therapeutic strategy for stroke recovery.

## Data Availability

No datasets were generated or analysed during the current study.

## Abbreviations

iPSC: induced pluripotent stem cell
KSR: KnockOut Serum Replacement
CDLC: chemically defined lipid concentrate
PFA: paraformaldehyde
MRI: magnetic resonance imaging
DAB: 3,3’-Diaminobenzidine Tetrahydrochloride
H&E: Hematoxylin and eosin
hNCAM: human-specific neural cell adhesion molecule
MVePC: parvocellular part of the medial vestibular nucleus
